# Association Between COVID-19 Vaccination and Abortion: A Cross-Sectional Study in Jeddah

**DOI:** 10.7759/cureus.33836

**Published:** 2023-01-16

**Authors:** Rehab A Alsaleh, Intessar Sultan, Jenan A Alasfour, Tarteel M Alaali, Amani S Alghamdi, Rehab A Mohammed

**Affiliations:** 1 Obstetrics and Gynecology, Ibn Sina National College for Medical Studies, Jeddah, SAU; 2 Internal Medicine, Ibn Sina National College for Medical Studies, Jeddah, SAU; 3 General Practice, Samir Abbass Hospital, Jeddah, SAU; 4 Medicine, Ibn Sina National College for Medical Studies, Jeddah, SAU; 5 Internal Medicine, Faculty of Medicine for Girls, Al-Azhar University, Cairo, EGY

**Keywords:** covid-19 in saudi arabia, saudi women, covid-19 vaccination, pregnancy, abortion

## Abstract

Background: Clinical trials for COVID-19 vaccines initially excluded pregnant women. However, observational studies revealed a relative safety of the vaccine during pregnancy therefore association between different types of COVID-19 vaccination and the risk of abortion must be studied.

Objectives: The objective is to explore the possible association between abortion and different types of COVID-19 vaccination in Jeddah.

Methods: This was a retrospective cross-sectional study done in three private general hospitals in Jeddah using electronic medical records and phone interviews of pregnant women who were admitted with abortion. Women were then interviewed for their vaccination data (type, dose) and their current pregnancy outcome (aborted or not).

Results: Medical records of 214 women diagnosed with abortion were included; 13.1% of them managed to continue their pregnancy. Vaccinated women (86%) had significantly earlier gestational age (p=0.031), higher hypertension (<0.001), and lower positive consanguinity (<0.001) compared to non-vaccinated women. The type (p=0.636) and number (p=0.331) of vaccination did not differ significantly among vaccinated women with and without abortion. Significant predictors of abortion were age>35 years (OR: 3.1, 95% CI: 1.34-6.97, p=0.008), diabetes (OR: 0.09, 95% CI: 0.01-0.89, p=0.040), and positive consanguinity (OR: 0.12, 95% CI: 0.02-0.63, p=0.012). However, spontaneous abortion did not have an increased odds of exposure to COVID-19 vaccines (OR: 1.07, 95% CI: 0.21-5.49, p=0.937).

Conclusion: COVID-19 vaccination is not associated with an increased risk of abortion in women vaccinated during their first or second trimesters. Further clinical trials are needed to support the evidence of the safety of early vaccination of pregnant women.

## Introduction

The Pfizer-BioNTech vaccine was the first vaccine to be approved for use in Saudi Arabia in December 2020 immediately after the publication of the Phase III clinical trial. Approval of the second vaccine, Oxford-AstraZeneca followed in February 2021 [1]. Later, other types of available vaccines were approved for continuing mass vaccination including pregnant women. For safety issues, the Saudi Food and Drug Administration and the ministry of health developed one electronic reporting platform for vaccine-related adverse events which is reviewed periodically by an expert panel [1].

Based on the current evidence, pregnant women are not among the vulnerable group getting COVID-19 infection [2] but if infected, they are at an increased risk of developing severe illness [3], miscarriage, and stillbirth [4], placental thrombosis [5] and preterm birth [6]. The presence of advanced maternal age, overweight, and comorbidities, mainly hypertension and diabetes further increase the risk of serious outcomes [2].

Vaccination against COVID-19 infection has been proven to be highly effective in preventing severe illness, hospital admission, and death among vaccinated adults including pregnant women [2]. The suggested rationale of the eligibility of pregnant women for vaccination relies on the fact that none of the authorized vaccines used live attenuated viruses [2]. However, the favorable benefit-risk profile of COVID-19 vaccines has not been documented in pregnant women [7]. The main reasons are the exclusion of pregnant women from most pivotal trials and the lack of comparison to the non-vaccinated control group. 

During the epidemic, there is a global effort by the CDC [8], ACOG [9], and other agencies [10,11] to include COVID-19 vaccination in the antenatal care of pregnant women despite the lack of safety data especially the long-term one [12]. Spontaneous abortion has been reported as an adverse perinatal outcome [13], a finding which was not consistent in other reports, especially for RNA-based types [14,15].

Because further studies are needed, this study aimed to explore the possible association between abortion and different types of COVID-19 vaccination among pregnant women who were admitted with the diagnosis of abortion in a private group of hospitals in Jeddah.

## Materials and methods

Study design

This was a retrospective cross-sectional study using electronic medical records and phone interviews. Clinical data sources were from three private general hospitals (Ibn Sina College Hospital, New Jeddani, and Alsafa Hospital) in Jeddah, Saudi Arabia.

Inclusion and exclusion criteria

The study included obstetric records of all pregnant patients admitted with abortion from January to December 2021. Their contact number was retrieved from the file, and they were interviewed by phone. Women who received their vaccination before pregnancy were excluded from the study. Incomplete electronic records or patients who were not willing to participate were excluded from the analysis.

Sampling

We collected records of 214 eligible cases over one year (from January to December 2021). According to G*Power 3.1.9.4 with effect size 0.5, alpha 0.05, and power 0.80., the sample size should not be less than 128 cases.

Data collection

Demographic, clinical, and obstetric data were extracted from files, while COVID-19 infection and vaccination data, and outcome (complete abortion or continuation of pregnancy) were taken over the phone as well as any missing obstetric data. The demographic data included age, nationality, and occupation. Obstetric data included parity and possible risk factors of abortion as age >35, smoking, positive consanguinity, history of abortion, and comorbidities (diabetes, hypertension, thyroid, renal, and autoimmune diseases). The type and the number of doses of COVID-19 vaccination, and whether the doses were similar or mixed were also collected.

Statistical analysis

Statistical data were analyzed using SPSS software version 22. Data were reported as the number and the frequency of categorical variables. For comparisons between vaccinated and non-vaccinated pregnant women and those who aborted and were not aborted, the χ2 test was used. Binary regression analysis was used to detect significant predictors of abortion among vaccinated women. All significant tests were two-tailed and were conducted at a minimum of 0.05 level.

## Results

After inclusion and exclusion criteria, medical records of 214 women who were admitted with abortion were included, and 13.1% of them continued their pregnancy. Most women (86%) received COVID-19 vaccination; 33.6% one dose, 52.3% two doses, 65.4% RNA-based, 9.3% Viral-Vector based, and 11.2% mixed type vaccination. Table 1 shows the characteristic data of admitted women with a diagnosis of abortion with and without vaccination. The predominant age group was from 25 to 35 years (57.9%) with primigravida constituting only 9.3% of the participants. Most women (90.7%) presented at an early gestational age (6-13 weeks). More than half suffered from missed abortion (55.1%), while the remaining had either incomplete (18%), threatened (13.1%), or inevitable (2.8%) abortion. Among the participants, 28 women managed to continue their pregnancy after receiving treatment at the hospital. Comparison between those vaccinated and not vaccinated revealed that vaccinated women presented significantly at earlier gestational age (p=0.031), had hypertension (<0.001), and lower positive consanguinity (<0.001) compared to non-vaccinated women (Table 1).

**Table 1:** Characteristic data of admitted women with the diagnosis of abortion (with and without vaccination).

		All women admitted with diagnosis of abortion N= 214 (86%)		Non-vaccinated women admitted with diagnosis of abortion N=30 (13.1%)		Vaccinated women admitted with diagnosis of abortion N=184 (86%)		P value
		N	%	N	%	N	%	
Age	19-25 years	26	12.1%	4	13.3%	22	12.0%	0.858
	25-35 years	124	57.9%	16	53.3%	108	58.7%	
	>35 Years	64	29.9%	10	33.3%	54	29.3%	
Parity	Primipara	20	9.3%	0	0.0%	20	10.9%	0.165
	Multipara	156	72.9%	24	80.0%	132	71.7%	
	grand multipara	38	17.8%	6	20.0%	32	17.4%	
Gestational Age	6-13 weeks	194	90.7%	24	80.0%	170	92.4%	0.031
	14-19 weeks	20	9.3%	6	20.0%	14	7.6%	
Type of abortion	Threatened	30	14.0%	2	6.7%	28	15.2%	0.141
	Inevitable	6	2.8%	2	6.7%	4	2.2%	
	Incomplete	60	28.0%	12	40.0%	48	26.1%	
	Missed	118	55.1%	14	46.7%	104	56.5%	
Spontaneous or induced abortion	Spontaneous	54	25.2%	8	26.7%	46	25.0%	0.531
	Induced	132	61.7%	20	66.7%	112	60.9%	
Abortion	No abortion	28	13.1%	2	6.7%	26	14.1%	0.261
	Abortion	186	86.9%	28	93.3%	158	85.9%	
Diabetes	No	204	95.3%	28	93.3%	176	95.7%	0.0577
	Yes	10	4.7%	2	6.7%	8	4.3%	
Hypertension	No	212	99.1%	2	6.7%	0	0.0%	<0.001
	Yes	2	0.9%	28	93.3%	184	100.0%	
Thyroid	No	212	99.1%	30	100.0%	182	98.9%	0.566
	Yes	2	0.9%	0	0.0%	2	1.1%	
Smoking	No	194	90.7%	30	100.0%	164	89.1%	0.058
	Yes	20	9.3%	0	0.0%	20	10.9%	
Consanguinity	Negative	158	73.8%	12	40.0%	146	79.3%	<0.001
	Positive	56	26.2%	18	60.0%	38	20.7%	
Hyperemesis	No	202	94.4%	30	100.0%	172	93.5%	0.150
	yes	12	5.6%	0	0.0%	12	6.5%	
Past history of abortion	1-2	68	31.8%	12	40.0%	56	30.4%	0.295
	>=3	10	4.7%	0	0	10	5.4%	
Number of risk factors of abortion	No risk factor	68	31.8%	4	13.3%	64	34.8%	0.065
	1 risk factors	76	35.5%	12	40.0%	64	34.8%	
	2 risk factors	50	23.4%	10	33.3%	40	21.7%	
	3 risk factors	14	6.5%	4	13.3%	10	5.4%	
	4 risk factors	6	2.8%	0	0.0%	6	3.3%	

Comparison between admitted women with complete abortion outcomes and those who continue their pregnancy (Table 2) showed that women with complete abortion had two significant risk factors age>35 years (0.012) and positive consanguinity (p=0.014) with no significant difference in their vaccination status (p=0.261), number of doses (p=0.331), type of vaccine (p=0.636), or timing (0.794).

**Table 2:** Comparison between admitted women with complete abortion outcomes and those who continue their pregnancy.

		All women admitted with abortion N=214				P-value
		No abortion n=28 (13.1%)		Complete Abortion N=186 (86.9%)		
		N	%	N	%	
Age	19-25 years	6	21.4%	20	10.8%	0.012
	25-35 years	20	71.4%	104	55.9%	
	>35 Years	2	7.1%	62	33.3%	
Parity	Primipara	2	7.1%	18	9.7%	0.230
	Multipara	24	85.7%	132	71.0%	
	grand multipara	2	7.1%	36	19.4%	
Gestational age	6-13 weeks	24	85.7%	170	91.4%	0.335
	14-19 weeks	4	14.3%	16	8.6%	
Diabetes	No	26	92.9%	178	95.7%	0.507
	Yes	2	7.1%	8	4.3%	
Hypertension	No	28	100.0%	184	98.9%	0.581
	Yes	0	0.0%	2	1.1%	
Thyroid	No	28	100.0%	184	98.9%	0.581
	Yes	0	0.0%	2	1.1%	
Smoking	No	28	100.0%	166	89.2%	0.068
	Yes	0	0.0%	20	10.8%	
Consanguinity	Negative	26	92.9%	132	71.0%	0.014
	Positive	2	7.1%	54	29.0%	
Vaccination	No	2	7.1%	28	15.1%	0.261
	Yes	26	92.9%	158	84.9%	
Number of vaccinations	One dose	8	28.6%	64	34.4%	0.331
	2 doses	18	64.3%	94	50.5%	
Type of vaccination	RNA-based vaccine	20	71.4%	120	64.5%	0.636
	Viral-Vector based vaccine	2	7.1%	18	9.7%	
	Mixed type	4	14.3%	20	10.8%	
Time of vaccination	First trimester	24	92.3%	148	93.7%	0.794
	Second trimester	2	7.7%	10	6.3%	

Gestational age, consanguinity, smoking, presence of comorbidities, and vaccination status all represented a significant model for the prediction of complete abortion (chi-square 27.96, p=0.001, Nagelkerke R Square 0.227). However, independent significant predictors were age>35 years (OR: 3.1, 95% CI: 1.34-6.97, p=0.008), presence of diabetes (OR: 0.09, 95% CI: 0.01-0.89, p=0.040), and positive consanguinity (OR: 0.12, 95% CI: 0.02-0.63, p=0.012), but not vaccination (OR: 1.07, 95% CI: 0.21-5.49, p=0.937) (Table 3).

**Table 3:** Predictors of complete abortion among all women admitted with abortion.

	P	OR	95% C.I for EXP(B)	
			Lower	Upper
Age	.008	3.061	1.344	6.971
Parity	.502	.706	.255	1.952
Gestational age	.537	.654	.170	2.515
Diabetes	.040	.089	.009	.891
Hypertension	.999	.000	.000	.
Thyroid	.999	.000	.000	.
Smoking	.998	.000	.000	.
Consanguinity	.012	.118	.022	.631
Vaccination	.937	1.068	.208	5.486

## Discussion

In this study, among 214 hospitalized pregnant women with the diagnosis of abortion; 13.1% managed to continue their pregnancy. Most of the participating women were vaccinated (86%) during their first or second trimesters with at least one vaccine, while 11.2% received mixed vaccines during pregnancy. The type and number of vaccinations did not differ significantly among vaccinated women with and without abortion. Significant predictors of abortion were the known risk factors of abortion as older maternal age, diabetes, and positive consanguinity but not the vaccination. These results support the absence of an association between the vaccine and abortion. The explanation may include significantly lower risk factors of abortion among the vaccinated group as younger age and less consanguinity. However, these results can still point to the safety of vaccination concerning the risk of abortion.

Many reports from observational studies particularly those using mRNA vaccination during pregnancy are in agreement with the current study. The study done by Shimabukuro et al. [7] reported the safety of the mRNA COVID-19 vaccine in pregnant persons based on the preliminary data from the “v-safe health checker” surveillance system, the v-safe pregnancy registry, and the Vaccine Adverse Event Reporting System. Moreover, one large study included more than 22,000 vaccinated women with at least one dose of the COVID-19 vaccine during pregnancy [16] and reported that neither the number of doses, the type, or the time of vaccination had any association with any risk even after comparison with the non-vaccinated group, or those vaccinated group after pregnancy.

Most of the admitted women with the diagnosis of abortion in this study were during their early pregnancy (90.7%) and they were significantly clustered in the vaccinated group (92.4%) but not in the group with complete abortion. In support of our findings, early abortion has not been associated with vaccination in two population-based large studies [15,17]. Moreover, many observational studies reported safe pregnancy outcomes after vaccination with mRNA COVID-19 vaccines late in the second or third trimesters [16-19]. Finally, in support of the general safety of vaccination during pregnancy, large observational studies did not detect significant neonatal or early infant poor outcomes among vaccinated pregnant women irrespective of their doses or timing of vaccination.

Strengths of this study include the retrieval of the high-risk group of pregnant women with threatened abortion who managed to continue their pregnancy similarly in both vaccinated and non-vaccinated groups.

This study has several limitations. First, the study relied on abortion data recorded in the medical records, therefore, selection bias is possible with the limitation of generalization of the results. Second, the observational study design limited the causal and effective relationship between abortion and the vaccination data. Third, there was no information on other vaccination as influenza vaccines which might represent a confounding factor.

## Conclusions

In this study, there were no significant associations between different types of abortion and COVID-19 vaccination among women who were vaccinated with either mRNA vaccines alone or mixed types of vaccines during their first or second trimesters. There is a need for better-designed clinical trials to support the evidence of the safety of all types and doses of COVID-19 vaccines given during earlier gestational age. Long-term data regarding the safety of the offspring are also needed.
